# Lifespan differences in emotional contagion while watching emotion-eliciting videos

**DOI:** 10.1371/journal.pone.0209253

**Published:** 2019-01-18

**Authors:** Ted Ruffman, Rebecca Then, Christie Cheng, Kana Imuta

**Affiliations:** 1 Department of Psychology, University of OtagoDunedin, New Zealand; 2 School of Psychology, University of Queensland, St. Lucia, Queensland, Australia; Swinburne University of Technology, AUSTRALIA

## Abstract

Previous research has examined empathic concern by presenting toddlers with a sad stimulus and examining their emotional response, with the conclusion that toddlers display empathy. Yet, such research has failed to include basic control conditions involving some other aversive stimulus such as white noise. Nor has it compared toddlers to adults to examine potential development in empathy. In the present study, we showed toddlers and adults four video types: infant crying, infant laughing, infant babbling, and a neutral infant accompanied by white noise. We then coded happiness and sadness while viewing the videos, and created a difference score (happiness minus sadness), testing 52 toddlers and 61 adults. Whereas adults showed more sadness towards infant crying than any other stimulus, toddlers’ response to crying and white noise was similar. Thus, the toddler response to crying was comparable to previous studies (slight sadness), but was no different to white noise and was significantly reduced relative to adults. As such, toddlers’ response seemed to be better characterized as a reaction to an aversive stimulus rather than empathy.

## Introduction

Research into children’s empathy tends to focus on two age groups: newborns and toddlers. The standard thinking is that newborns are capable of a primitive form of empathy, emotional contagion, which then develops into a more full-blown form of empathy in toddlers (see below). Nevertheless, some have recently questioned whether the neonate reaction to crying is truly indicative of empathy, and in the present study, we examined whether toddlers’ reaction to crying is truly empathic by (a) comparing crying to white noise (to determine whether the toddler crying response simply represents a response to an aversive stimulus), and (b) comparing toddlers to adults (to determine whether adults show a clearer empathic reaction to crying than toddlers). Thus, we summarize research for newborns and toddlers below.

### Neonates

For nearly 50 years, the conclusion has been that neonates have a basic form of empathy: emotional contagion. In experiments with neonates, an infant’s spontaneous cry has been recorded and played for other infants, alongside other decibel-matched aversive sounds such as the infant’s own cry, a primate cry, or white noise. The general finding is that neonates cry more quickly when listening to other neonates cry relative to their own cries, older infant cries, monkey cries or white noise [1–3]. These findings have led many researchers to confidently state that newborns possess a basic form of empathy [4].

However, researchers recently challenged the idea that contagion and empathy are innate [4]. One of the perplexing findings is that neonates cry when hearing another newborn’s cry, but not when hearing an older infant cry. In contrast, adults respond with empathy when they hear the cries of an older infant [5]. If adults perceive suffering in older infant cries, and neonates are truly empathic, then one would think that neonates should also respond to older infant cries with sadness. Yet, Martin and Clark [1] found that neonates who were initially calm, cried substantially more when listening to another neonate cry compared to an older infant cry. Such findings suggest that neonates’ crying response to a newborn cry might stem from something other than empathy. That is, newborns might find the cries of another newborn aversive, not because a newborn’s cry conveys sadness, but because the acoustic properties of a newborn cry are distinct from older infant cries, and more aversive than the acoustic properties of older infant cries, therefore causing greater discomfort in a newborn infant. Certainly, there is evidence for distinctness in that newborn cries have a lower fundamental frequency (pitch) than older infant cries [6, 7].

There is also ample evidence that certain features of sounds are emotionally aversive *even when they don’t convey emotion*. Kumar, Bailey, and Griffiths [8] asked adult participants to rate sounds on a 0 to 9 scale. A knife scraping on a bottle was rated as the most aversive (*M* = 8.10), with a baby’s cry also aversive but rated 25^th^ of 75 sounds and substantially lower (*M* = 5.92). In general, sounds with a higher frequency and lower temporal modulation were perceived as more aversive. In other words, sounds that *do not express emotion* (e.g., knife on bottle) are often rated as very aversive. Further, sounds that don’t express emotion can create an emotional reaction in a listener. Reuter and Oehler [9] found that the sound of fingernails on a blackboard led to increased sweating and skin conductance in listeners. Likewise, all sounds are first processed in the auditory cortex (superior temporal gyrus) [8], but particularly unpleasant sounds (e.g., knife on bottle) lead to more connected activation from the amygdala to the superior temporal gyrus, with the amygdala having “a central role in emotional phenomenology” [10]. In sum, aversive sounds that do not express emotion can create an emotional reaction, making it unclear whether neonates respond to the suffering neonate crying conveys or to the acoustic properties of neonate crying, which are aversive irrespective of any suffering conveyed.

### Toddlers

By the time children are toddlers, empathy is sometimes tested using helping paradigms. Generally, three kinds of helping are distinguished: instrumental helping (e.g., helping someone who has dropped an object but does not emote), empathic helping (e.g., helping someone who is distressed), and altruistic helping (e.g., giving up something of one’s own to help someone who is distressed). In general, instrumental helping tends to evolve earliest, followed by empathic helping, and then altruistic helping [11]. For instance, 18-month-olds engage in instrumental helping [12].

Another paradigm measures pupil dilation in addition to helping behaviour. For instance, 25-month-olds’ pupil dilation is larger when an agent in need of help receives no help relative to when either the child or another person helps the agent, with pupil dilation thought to signify empathy [13]. Similarly, 2-year-olds help an adult by retrieving an object the adult needs (but not one the adult didn’t need), and children with greater internal arousal as measured by pupil dilation, are faster to help [14]. In a second experiment, they also found that children’s pupil size increased when they witnessed an adult respond inappropriately to another’s need by retrieving the incorrect object. In contrast, in a non-social condition, when an object flew to the adult on its own, children’s pupil size did not increase.

These results are consistent with the idea that children empathically try to help others, and respond empathically (signalled by pupil dilation) when another needs help. However, they are also consistent with alternative explanations. For instance, children might help more out of expectation rather than empathy, having learned that they tend to be castigated when they do not meet expectations and praised when they do (just as they learn to say “please” and “thank you”). Thus, they might help, not out of empathy, but in order to maximize positive outcomes for themselves. Likewise, pupil dilation can indicate surprise [15] or general arousal [16] rather than empathy. To this end, it is plausible that children learn to expect certain behaviours on the basis of their observations in the world [17] and are subsequently surprised when an adult brings the wrong object [13]. Further, it is likely that children’s arousal (and pupil dilation) increases when anticipating a likely outcome that an adult will aid another by bringing a needed object, yet no such outcome eventuates [14]. These explanations account for helping and pupil dilation without invoking empathy.

In addition, empathy in toddlers is frequently tested by measuring empathic concern (facial responses) when viewing another’s suffering (e.g., following an accident or when listening to or watching an infant crying). In such studies, empathy is sometimes rated as present versus absent, and although the findings are typically interpreted as indicating toddlers are empathic, the reality is that the majority of children do not express empathic concern. For instance, only about ¼ of toddlers show empathic concern and only about ½ act prosocially [18, 19]. Many children are simply unresponsive (35% of 14-month-olds and 25% of 20-month-olds), even when as many as five distress stimuli are presented [20].

Furthermore, empathic concern is frequently rated on a scale. For instance, 18- and 24-month-olds were rated as experiencing more empathic concern and distress when listening to a crying infant than a neutral infant [21]. Typically, the mean rating for children will lie above some minimum value, yet also, well below the maximum. For instance, in Zahn-Waxler, Robinson, and Emde [20], there was a mean of 2.45/4.00 for 24-month-olds [21–24]. The question thus becomes whether a rating of 2.45/4.00 represents an empathic response, with three issues paramount.

First, in this rating scale (and the scales of many others), “1” represents no response and “2” represents a “fleeting or slight change of expression that includes brow furrow” [20]. The question is, “Is a fleeting brow furrow a genuine expression of empathy?” For instance, in one study, older adults’ brow furrowing when presented with angry emotion faces was an expression of *confusion* rather than empathy, in that more brow furrowing was associated with *worse* recognition of anger [25]. Infant brow furrowing could, likewise, represent confusion rather than empathy. Second, whether empathic concern is rated via helping response, presence/absence of facial expression, or rating of facial expression, in all cases such studies report group means. This leaves open the possibility that fewer toddlers would show empathic concern (e.g., brow furrowing) compared to an older age group (e.g., adults). In other words, even if a proportion of toddlers are empathic, it is important to examine the extent of age-related development subsequently. Third, unlike research with neonates, in research with toddlers, both when using a rating scale and when rating empathy as present/absent, researchers have not used control stimuli such as white noise to compare to crying/distress. This might be due to the widely held assumption that empathy is present by the age of 2 years, within the first year [26, 27], or even in neonates (see above), which has likely led to less stringent methods for demonstrating that toddlers are empathic. However, because a child’s unhappiness could stem from either empathic concern regarding another’s suffering *or* the acoustic properties of a crying stimulus (which are acoustically aversive), this is a crucial omission.

One way of helping to adjudicate between these possibilities is to examine correlations with other abilities or behaviors that might indicate genuine empathy. In general, there is some evidence of such relationships. Toddlers who show empathic concern (a) also tend to show helping behaviour [18–20, 24], and (b) acquire emotion words at a faster rate, even after accounting for general language [22]. Nevertheless, empathic concern does not always correlate with relevant abilities. For instance, whereas toddlers’ *helping behavior* correlates with self-recognition [18, 28], it is less clear whether empathic concern does so also. Nichols et al. [22] found a correlation by measuring children’s self-recognition via maternal questionnaire, but when using actual recognition tasks [18, 29], there is no relation. In addition, when correlations between empathic concern and other relevant abilities do exist they tend to be modest, once again leaving open the possibility that at least some children who are rated as showing empathic concern are responding to the acoustic characteristics of a stimulus.

### Present study

In the present study, we overcame such ambiguities in interpretation by comparing participants’ responses toward a video of infant crying to a white noise control condition (a video of an infant with a neutral expression, accompanied by white noise). As far as we are aware, previous researchers examining empathy in toddlers have not included such a control condition when examining empathic concern. However, if toddlers did not show a differential response to crying versus white noise, one could argue that videoed infants are generally unsuccessful in eliciting any kind of toddler emotional reaction. Thus, we also included two other control videos–infant laughing and infant babbling. Of interest was whether toddlers might experience contagious smiling when viewing laughing relative to babbling. Contagious smiling would demonstrate sensitivity to the emotional content of the videos even if toddlers did not differentially respond to crying versus white noise.

We also examined whether empathy continues to develop beyond the toddler years by comparing toddlers to adults. That is, even if toddlers show signs of empathy, it is important to examine potential limitations in their empathic concern. Once again, we are not aware of any previous studies that have made this comparison but empathic concern is likely to evolve gradually, hence highlighting the usefulness of a comparison to adults.

When watching all four videos, participants themselves were also videoed, and the videos of the participants were subsequently coded for participant happiness and sadness. For each video, we then created a difference score (happiness rating minus sadness rating), hypothesizing that the laughing videos would produce the most happiness, followed by babbling, white noise, and crying. Empathy would potentially be indicated if toddlers showed less overall happiness (i.e., more in the way of sadness) when viewing the crying video relative to the white noise video.

In addition, in preliminary analyses within our adult group, we directly compared the responses of young and older adults. We did not necessarily anticipate differences between young and older adults because research indicates that empathic concern is similar in young and old [30, 31]. Nevertheless, with only two relevant studies to date, we examined older adults’ empathic responses further, although because our main interest was in toddler empathy rather than young versus older adult empathy, we obtained a larger sample of toddlers compared to the young and older adult groups, with the expectation of eventually collapsing the two adult groups (assuming equal empathic concern) to form a single adult group of comparable size to the toddler group.

In sum, we had three main aims: (1) comparing toddlers’ response to crying to that of adults, (2) comparing responses to crying and white noise within age groups, and (3) examining responses to video stimuli in general using infant laughing and babbling.

## Materials and methods

This study was approved by the University of Otago Human Ethics Panel, F17/008. Parents of children and adults gave informed, written consent to participate.

### Participants

The participants included 56 2-year-olds (toddlers) drawn from working- and middle-class homes. Four toddlers were excluded due to inattention, leaving a remaining total of 52 (*M* = 2.53 years, range = 24 to 36 months, 26 females). Mothers expressed an interest in participating in experiments upon the birth of their child and were subsequently phoned to gauge their interest in the present study. They then came to the Psychology department for testing and were given a $10 petrol voucher to cover travel costs. Children were given a small gift upon completion of the study. Mothers and children were from a wide range of socio-economic (e.g., educational) backgrounds. In addition, there were 32 young adults (*M* = 20 years, range = 18–27 years, 22 females) and 29 older adults (*M* = 75 years, range = 65 to 85 years, 17 females). The young adults were first- and second-year psychology students who completed a written assignment after the experiment for a small contribution to their course grades. The older adults were volunteers from the community who were given a petrol voucher to cover their travel costs. Whereas all young adults were in their first or second year of university study, older adults had a wide range of educational achievement, ranging from some high school through to post-graduate degrees.

As a screening device, adult participants were given the Matrices task of the Weschler Adult Intelligence Scale IV to measure fluid intelligence. Healthy aging is associated with a decline in fluid IQ relative to young adults [32]. Five young adults and four older adults did particularly poorly on this measure (more than 2 *SD*s below their respective group means). In the older adults, lower fluid IQ scores are not unexpected and are likely associated with a more advanced process of aging. In contrast, particularly low fluid IQ scores in young adults (as low as the lowest scoring older adults) were unexpected and are more likely due to inattentiveness. Indeed, research has questioned the motivation of typical undergraduate samples [33]. Thus, as a conservative measure, we omitted the five young adults who performed particularly badly on the fluid IQ measure from the main analyses.

### Videos

There were 20 different videos in total, each featuring a different infant, with five each of crying, white noise, laughing and babbling. Crying infants were very unhappy with near-continuous audible crying. Laughing infants were very happy with near-continuous laughing. Babbling infants had a neutral facial expression and babbled near continuously. Neutral infants had a neutral expression and produced no audible sound. The videos were looped to each be one-minute long, with each averaging 70dB. Infants were typically featured face-on and close-up. Each participant was presented with a set of four video clips (one of each type), meaning there were five video sets in total, with participants assigned in roughly equal numbers to each video set. The order of the videos in each set was randomized, as was the video set that was given to each participant.

Videos were obtained from Youtube using the search terms, “sad crying infant”, “happy laughing infant”, “babbling infant” and “infant”, and featured both young and older infants. The white noise was recorded with an amplifier and microphone, and was then manipulated to have similar frequency modulations (pitch rising and falling) and mean intensity to the crying, babbling and laughing stimuli (with 10 white noise clips created in total). Example videos can be provided by the first author.

### Procedure

Adults and parents of toddlers visited the psychology laboratory (a small wooden house on the university campus) and were told simply that we were interested in how people responded to different kinds of videos. Adults sat one metre from the computer screen and a video camera behind the screen recorded their facial expressions as they watched. For children, caregivers sat on a chair that was situated in front of a computer screen with the child on the caregiver’s lap one metre from the screen. The caregiver was told not to interact with the child or show any emotional expression throughout the experiment and wore a blindfold and noise-cancelling headphones. The blindfold ensured that the caregiver could not see what the child was watching and the headphones played classical music to drown out the sound of the video clips. All participants were left alone in the room when viewing the videos, with the experimenter returning when she could hear that the videos had finished.

For each video, we coded head turns away from the computer monitor as well as each participant’s emotional response. Head turns were much more frequent in toddlers but were not different over conditions, likely because they could represent many different things (sharing of the content with mother, boredom, aversion). Our main interest was in the participant’s facial reactions over the 60-s recording, which was split into 20 3-s time bins, with each time bin coded separately. For each time bin, we used a 5-point Likert scale, to separately code facial expressions of happiness and sadness. For laughing, a rating of 1 was coded as no emotional expression, 3 as a moderate, steady expression (e.g., a sustained smile for the happiness rating), and 5 as an extreme, intense expression (e.g., continuous laughter for the happiness rating). For sadness, 1 was coded as no emotional expression, 3 as a sustained sad expression, and 5 as an extreme, intense expression (e.g., continuous sadness with tears in eyes). Happiness and sadness were coded separately because a participant could conceivably express both within the same time bin. There were two coders, both blind to the video children watched. One coder was assigned to code all of the videos while the second coded 50%. The main outcome variable in our analyses was a happiness difference score (mean happiness rating over the 20 time bins–mean sadness rating over the 20 time bins). We used this difference score because it took into account overall happiness/sadness in each time bin unlike a rating of happiness or sadness on their own. The intra-class correlation coefficient for the happiness difference score, indicating reliability between the two coders, was, *α* = .81, a level of alpha regarded as ‘excellent’ [34].

## Results

In this section we first compare young adults to older adults, then compare toddlers to adults as a group. Finally, we consider individual performance rather than group performance.

### Preliminary analysis: Young adults versus older adults

**Fig 1:**
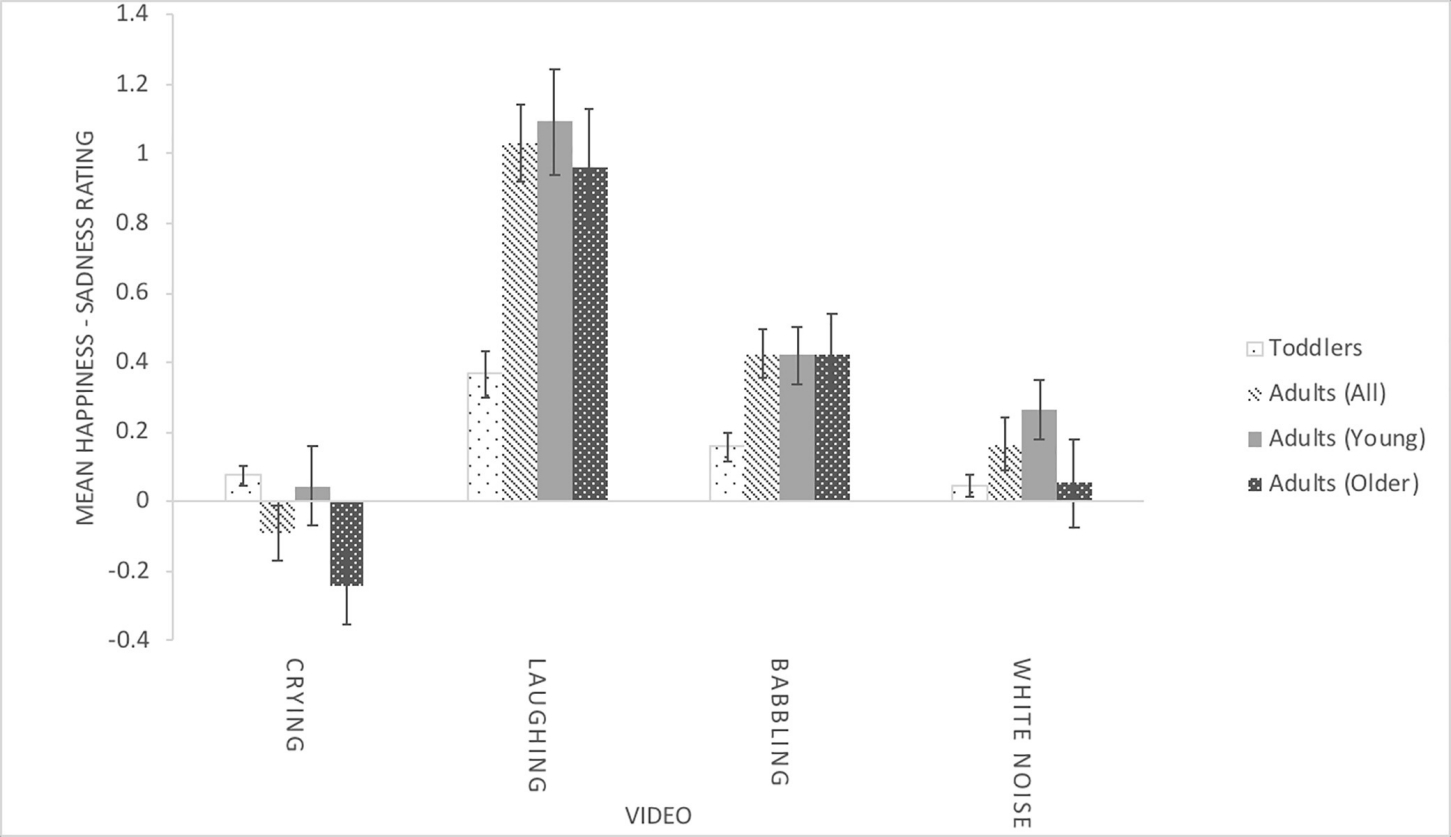
Mean happiness minus Sadness Difference scores (SD).

Fig 1 shows the descriptive statistics for the happiness difference score (hereon, ‘happiness score’) for the different videos and for each age group. In the first analysis, we compared young and older adults’ happiness scores using a 2 (Age Group) x 4 (Video) analysis of variance (ANOVA). Age Group was a between-subjects variable, Video was a within-subjects variable, and happiness score was the dependent variable. For all ANOVAs, the Greenhouse-Geisser correction was used when there was a violation of sphericity. The interaction was of particular interest and, if significant, would indicate that young and older adults responded differently to the videos. The only significant effect was for Video, *F*(2.33, 137.59) = 33.83, *p* < .001, *η*_*p*_^*2*^ = .364. The effect for Age Group was not significant, *F*(1, 59) = 2.93, *p* = .092, *η*_*p*_^*2*^ = .047, and neither was the interaction, *F*(2.33, 137.59) = 0.56, *p* = .600, *η*_*p*_^*2*^ = .009. We then eliminated the five young adults with particularly low fluid IQ scores because of likely inattentiveness [33] and again compared young and older adults using a 2 x 4 ANOVA. The result was the same. There was a main effect for Video, *F*(2.23, 120.48) = 29.56, *p* < .001, *η*_*p*_^*2*^ = .354, whereas the effect for Age Group was not significant, *F*(1, 54) = 1.66, *p* = .203, *η*_*p*_^*2*^ = .047, and neither was the interaction, *F*(2.23, 120.48) = 0.38, *p* = .710, *η*_*p*_^*2*^ = .007.

Because there were no effects for age group and no interaction between age group and video, we collapsed across young and older adults to form one adult age group. For the remaining analyses, we eliminated the five young adults with particularly low fluid IQ scores, just as we had also eliminated four toddlers at the outset due to inattentiveness, in order to obtain results maximally representative of participants’ true abilities. However, we note that an identical pattern was obtained if the five young adults with particularly low scores on the fluid IQ task were retained in the analyses.

### Toddlers versus adults

In the next analysis, we compared toddlers to adults using a 2 (Age Group: toddlers, adults) x 4 (Video) ANOVA. All effects were significant, including the effect for Video, *F*(2.23, 235.96) = 39.35, *p* < .001, *η*_*p*_^*2*^ = .271, the effect for Age Group, *F*(1, 106) = 12.83, *p* = .001, *η*_*p*_^*2*^ = .108, and the interaction, *F*(2.23, 235.96) = 12.29, *p* < .001, *η*_*p*_^*2*^ = .104.

The interaction was of most interest and was first explored with *t*-tests comparing each pair of video conditions. Conditions were compared separately in each age group and Holm’s correction used to ensure the family-wise error rate was *p* < .05. Of particular interest was whether there was a difference in happiness when watching the crying video versus the white noise video. Toddlers looked significantly happier when viewing laughing compared to crying, *t*(51) = 4.05, *p* < .001, Cohen’s *d* = .561, laughing compared to white noise, *t*(51) = 4.49, *p* < .001, Cohen’s *d* = .622, and laughing compared to babbling, *t*(51) = 3.39, *p* = .001, Cohen’s *d* = .471. Clearly, then, laughter was contagious for toddlers as a group and the videos were generally able to elicit emotion in toddlers. However, no other effects reached significance after using Holm’s correction, including babbling versus white noise, *t*(51) = 2.41, *p* = .020, Cohen’s *d* = .335, crying versus babbling, *t*(51) = 2.20, *p* = .032, Cohen’s *d* = .307, and most importantly, crying versus white noise, *t*(51) = 0.73, *p* = .471, Cohen’s *d* = .101. We then used a Bayes analysis to determine whether this lack of a difference in toddlers’ response on crying versus white noise represented a genuine null effect. In this case, the Bayes factor was .09, with any value less than .33 indicating acceptance of the null hypothesis of no difference [35]. Thus, we accept that toddlers’ emotional reaction to crying was no different than to white noise.

Next, we examined adults. Adults looked significantly happier when viewing laughing compared to crying, *t*(55) = 7.19, *p* < .001, Cohen’s *d* = .960, laughing compared to white noise, *t*(55) = 6.02, *p* < .001, Cohen’s *d* = .805, laughing compared to babbling, *t*(55) = 4.96, *p* < .001, Cohen’s *d* = .662, babbling compared to crying, *t*(55) = 4.66, *p* < .001, Cohen’s *d* = .623, and babbling compared to white noise, *t*(55) = 2.59, *p* = .012, Cohen’s *d* = .346. Most importantly, adults looked significantly happier when viewing white noise compared to crying, *t*(55) = 3.09, *p* = .003, Cohen’s *d* = .414. Thus, the most important difference between toddlers and adults was that toddlers showed the same amount of happiness/sadness when viewing the white noise and crying videos, whereas adults looked significantly less happy when viewing the crying video.

We then used a second means of exploring the Video x Age Group interaction by comparing toddlers’ and adults’ happiness scores in each of the four videos. Adults showed significantly more happiness than toddlers when viewing the laughing videos, *t*(108) = 4.78, *p* < .001, and the babbling videos, *t*(108) = 3.20, *p* = .002. Adults also showed significantly less happiness than toddlers when viewing the crying videos, *t*(108) = 2.34, *p* = .021. There was no difference on the white noise videos, *t*(108) = 0.75, *p* = .458.

One could argue that toddlers might have shown both more sadness and more happiness while watching crying and the two responses might have cancelled each other out. Thus, we examined ratings of sadness on their own using a 2 (Age Group: toddlers, adults) x 4 (Video) ANOVA, with sadness rating as the dependent variable. As for the difference score, there was a main effect for Video, *F*(2.03, 215.31) = 7.87, *p* < .001, *η*_*p*_^*2*^ = .069, a main effect for Age Group, *F*(1, 106) = 6.72, *p* = .011, *η*_*p*_^*2*^ = .060, and a significant interaction, *F*(2.03, 215.31) = 3.83, *p* = .010, *η*_*p*_^*2*^ = .035. We examined the interaction with four independent-samples *t*-tests comparing toddlers and adults during each video, and using Holm’s correction. Adults showed more sadness when viewing crying than toddlers, *t*(106) = 2.70, *p* = .008, Cohen’s *d* = .546 (Toddlers: *M* = 1.05, *SD* = 0.11; Adults: *M* = 1.25, *SD* = 0.51), whereas there was no difference in sadness when viewing white noise, *t* = 1.36, *p* = .177, Cohen’s *d* = .266 (Toddlers: *M* = 1.05, *SD* = 0.11; Adults: *M* = 1.13, *SD* = 0.39), babbling, *t* = 1.27, *p* = .208, Cohen’s *d* = .242 (Toddlers: *M* = 1.00, *SD* = 0.02; Adults: *M* = 1.02, *SD* = 0.10), or laughing, *t* = 0.27, *p* = .786, Cohen’s *d* = .052 (Toddlers: *M* = 1.03, *SD* = 0.10; Adults: *M* = 1.03, *SD* = 0.13).

### Women versus men

One possibility is that adults showed more empathy when viewing a crying infant compared to white noise because there was more of a social expectation on adults than toddlers to be empathic. If correct, women should have demonstrated this tendency to a greater extent that men because empathy is much more central to the female gender role than the male gender role [36]. To examine this possibility, we compared women versus men in S1 File. There was no indication that gender (or social expectation) played a role in determining adult responding.

## Discussion

As summarized above, previous research indicates that some toddlers display unhappiness when viewing or hearing someone suffering. Such unhappiness has been argued to convey empathic concern because it correlates with empathic helping, the acquisition of emotion words, and sometimes with self-recognition. Yet, we pointed out that there are several reasons to remain cautious. First, the number of toddlers who show empathic concern is not high (around ¼), leaving open the possibility for age-related development in empathy, and that adults would be more empathic than toddlers. Second, not all studies obtain correlations between empathic concern and other behaviors, and when they are obtained they tend to be modest. Third, previous researchers examining empathy in toddlers have not employed control conditions (e.g., white noise) to determine whether children’s unhappiness is a reflection of empathy or angst due to an aversive auditory stimulus. In sum, it was possible that (a) toddlers would not react differently to crying and white noise, in which case it would seem that their response to crying was not empathy, (b) more adults than toddlers would show the expected pattern of emoting when viewing crying (less happiness than sadness), thereby revealing further limitations in toddlers’ empathic responding, and (c) adults who showed the expected pattern would show it more strongly than toddlers.

We examined such issues by presenting toddlers and adults with four types of videos: infant crying, infant laughing, infant babbling, and a neutral infant accompanied by white noise. There were three main aims: (1) comparing toddlers’ response to crying to that of adults, (2) comparing responses to crying and white noise within age groups, and (3) examining responses to video stimuli in general using infant laughing and babbling to ensure that toddlers were responsive to video stimuli.

Our findings were as follows: (a) adults but not toddlers showed less happiness and more sadness when viewing crying than white noise, (b) there was no difference in adults’ versus toddlers’ reaction to white noise, but adults showed less happiness than toddlers when viewing crying, (c) both toddlers and adults showed more happiness when viewing laughing compared to any other video, (d) adults but not toddlers showed more happiness when viewing babbling than crying or white noise, and (e) young and older adults did not differ in response. Further, the analyses for individuals indicated that (f) more adults than toddlers showed the expected pattern of emoting, and (g) adults who showed the expected pattern showed it more strongly than toddlers who did so.

As stated above, we are not aware of previous comparisons between toddlers and adults, but our findings suggest that toddler empathic concern is limited relative to adults. Indeed, the results of the present study, on their own, provide no evidence whatsoever for empathy in toddlers; because toddlers’ response to white noise was identical to that for crying, their response can be more parsimoniously described as a reaction to an aversive auditory stimulus rather than empathy. However, before accepting this conclusion an important issue is whether our methodology examining an emotional response to a sad stimulus was ideal for eliciting an empathic response. In fact, we used the same methodology as many other researchers (see above) and our results for crying were similar in that many participants (42% of toddlers and 45% of adults) showed no emotional reaction at all to the crying videos, and those who did showed only a mild reaction (with overall means only marginally above 1, “no response”). We note that children can show a range of responses to others’ distress, including a facial expression of sympathy/sadness, other-focussed distress (bodily and facial tension such as pressed lips but continued focus on distressed other), and self-focussed distress (similar to above but avoidance of other), with only sympathy/sadness a consistent correlate of helping behavior [37, 38]. Thus, children in our study showed the milder form of response associated with empathic responding, making both our method and our findings for crying similar to previous studies. The major points of departure were (a) the inclusion of the white noise control and toddlers’ similar responding for crying and white noise and (b) the inclusion of the babbling and laughing controls, which showed that the similar response to crying and white noise was not due to a general failure for toddlers to respond to the emotional content of videos.

Of crucial interest, therefore, is how to explain our finding that toddlers responded similarly to the crying and white noise videos. One possibility is that toddlers may have been empathic but were generally inexpressive or inexpressive due to self-consciousness. Yet, such arguments are inconsistent with common sense in that toddlers would typically be construed as generally less inhibited and more expressive than adults, and self-consciousness seems much more applicable to adults than toddlers. Second, toddlers might have been puzzled by the relatively novel expression of sadness (compared to happiness) and therefore reacted less intensely than adults. However, if correct, this explanation implies no empathy or understanding of sadness in toddlers because confusion does not entail either. Further, we note that white noise was also novel and, therefore, should have been equally puzzling for toddlers, and if so, should have led toddlers to also show less sadness to white noise than adults, yet toddlers and adults responded similarly to the white noise stimulus. Therefore, the most plausible explanation of the present results would seem to be that toddlers did not show clear signs of empathy.

This conclusion is clearly at odds with the consensus view discussed at the outset that toddlers and even newborns are empathic. Given this clear discrepancy between the consensus view and the present results, we again consider the evidence for each view. On the pro-empathy side, toddlers’ empathic concern correlates with various other tendencies (self-recognition, emotion word acquisition, helping behavior) suggesting genuine understanding. On the other hand, there is a lack of a credible control condition in previous research with toddlers, the frequent finding that only a minority of toddlers respond empathically, and the fact that correlations with other abilities are not always obtained and tend to be modest when they are (see above). Further, in our study, there is the fact that toddler unhappiness when viewing crying was identical to that when exposed to white noise, that a Bayes analysis indicated that we could accept the null hypothesis of no difference in toddler unhappiness to crying and white noise, that despite there being no difference in toddlers’ response to crying and white noise they were not generally unresponsive to the video stimuli (i.e., they responded with more happiness to laughing compared to the other three video types), and the fact that adults showed a different pattern with more sadness to crying than white noise. In short, although it might appear plausible that toddlers are empathic, previous studies with toddlers seem to have been guided by implicit assumptions of empathy and have, therefore, not included basic control conditions such as white noise. In consequence, they have not provided strong evidence for toddler empathy, and the present findings question basic assumptions about toddler empathy. For instance, it would have been more convincing if previous studies had employed white noise control conditions and showed that toddler unhappiness on these conditions does not correlate with abilities thought to measure empathy.

What, then, leads toddlers to help others? Toddler helping is more likely when the helpee gives explicit communicative cues regarding the need for help [39], when mothers talk about emotions [40], when parents socialize prosocial behavior [41], and when parents have stressed the importance of kindness to others [42]. Similarly, parental talk about emotions and mental states has been shown to relate to preschoolers’ theory of mind [43], and to children’s cooperation with others [44]. In addition, in a large meta-analysis, there was a modest correlation between theory of mind and prosocial behaviour (*r* = .17) [45]. Thus, the findings converge on the idea that parental emphasis on the importance of kindness and helping, clear information that someone needs help, and theory of mind will encourage toddlers to help others and prosocial behavior more generally.

It is important to consider reasons for why toddlers failed to respond differently to crying and white noise. One possibility is simply that we lacked statistical power to uncover a difference. However, (a) with identical power, we obtained a difference between toddlers’ response to laughing and the other videos, (b) we used Bayes test that allowed us to accept the null hypothesis of no difference in toddler responding to crying and white noise, and (c) with a similarly-sized sample, we obtained clear differences between crying and white noise in adults. A second possibility is that toddlers’ response to crying did indeed signal empathy but that they found white noise particularly aversive, something adults might grow out of. Once again, the data are not consistent with such an argument because although adults were significantly less happy when viewing crying relative to toddlers, there was no difference in toddler and adult responses to white noise. Thus, our data are more in line with the idea that empathy to crying increases with age, whereas the response to white noise does not change.

Nevertheless, our findings do suggest an avenue for future research. This could include both a crying and white noise condition, and examine whether toddler unhappiness in the crying condition is more highly correlated with emotion word acquisition/self-recognition than is toddler unhappiness in the white noise condition. Inclusion of a white noise control, and such a selective pattern of correlations, would provide clearer evidence that toddlers respond empathically to others’ suffering. Examining changes in response over time in a longitudinal study (e.g., between 9 months and 48 months of age) would help to indicate whether children’s response changes during development. Further, examining the suffering of different agents (e.g., infant versus adult) would elucidate whether children respond empathically to adults even if they don’t do so to infants.

The present study also sheds light on the general phenomenon of contagion. Both toddlers and adults showed evidence of contagion in their response to infant laughter in that they looked happier when viewing the laughing video than any of the other three videos (although adults showed a significantly stronger response to laughing compared to toddlers). Contagious laughing is largely an under-studied phenomenon, although there is some evidence of contagious laughter in adults [46], contagious glee in a small sample of children (*N* = 20) aged 29 to 65 months [47], and that 2- to 4-year-olds laugh more in the presence of a laughing adult compared to a non-laughing adult. Yet, in these prior studies it is not clear whether children laughed due to contagion, or whether the presence of laughing others created a social demand to also laugh to gain acceptance. In the present study, each child watched the videos while sitting on her/his mother’s lap such that the child could not have responded to maternal cues because mothers were blindfolded, wore headphones to cancel out auditory cues in the video, and therefore, could not see or hear the videos or their children, thereby reducing the social demand to laugh. Although toddler laughing does not require them to have conscious insight into another’s state of mirth, it nevertheless shows that toddlers responded to aspects of the emotional content conveyed in the videos, thereby making their failure to respond differently to crying and white noise all the more notable.

## Conclusions

In sum, the present study demonstrated age-related development in empathic concern; whereas adults provided clear evidence for empathy, toddlers did not. Although it seems plausible that some toddlers might experience empathic concern, we think that strong conclusions await the results of future research that employs relevant control conditions and examines developmental changes through early childhood and into adulthood.

## Supporting information

S1 Dataset (SAV)

S1 File Analysis of men versus women. (DOCX)
