# Supplementary material for: Lifespan differences in emotional contagion while watching emotion-eliciting videos
Source: PLoS One. 2019 Jan 18;14(1):e0209253. doi: 10.1371/journal.pone.0209253 (PMC6338362; doi:10.1371/journal.pone.0209253)
Supplement: S1 File — (DOCX) [file pone.0209253.s002.docx]

**Supplementary Materials**

**Supplementary Materials**

**Women versus men**

One possibility is that adults showed more empathy when viewing a crying infant compared to white noise because there was more of a social expectation on adults than toddlers to be empathic. If correct, women should have demonstrated this tendency to a greater extent that men because empathy is much more central to the female gender role than the male gender role [48]. To examine this possibility, we used a 2 (Gender: women, men) x 4 (Video) ANOVA, with the happiness difference score as the dependent variable. Of key interest was the interaction between video and gender, which should have been significant if women demonstrated more empathy than men when viewing a crying baby. There was a main effect for Video, *F*(2.22, 119.79) = 26.28, *p* < .001, *η_p_^2^* = .333, but not for Gender, *F*(1, 54) = 1.75, *p* = .192, *η_p_^2^* = .031, nor for the interaction, *F*(2.22, 119.79) = 0.54, *p* = .600, *η_p_^2^* = .010. Thus, there was no indication that social expectation played a role in facial response when viewing the crying versus the white noise videos.

**Reference**

48. Christov-Moore L, Simpson EA, Coudé G, Grigaityte K, Iacoboni M, Ferrari PF. Empathy: Gender effects in brain and behaviour. Neurosci Biobehav Rev. 2014;46: 604-627.
